# Aortic Valve Replacement in Severe Factor V Deficiency and Inhibitor: Diagnostic and Management Challenges

**DOI:** 10.7759/cureus.5918

**Published:** 2019-10-15

**Authors:** Alexa Bello, Eric Salazar, Kirk Heyne, Joseph Varon

**Affiliations:** 1 Research, Dorrington Medical Associates, Houston, USA; 2 Pathology and Genomic Medicine, Houston Methodist Hospital, Houston, USA; 3 Medical Oncology and Hematology, Houston Methodist Hospital, Houston, USA; 4 Critical Care, United General Hospital, Houston, USA

**Keywords:** factor v deficiency, prothrombin time, activated partial thromboplastin time, normal thrombin time, aortic valve replacement, fresh frozen plasma.

## Abstract

Factor V (FV) deficiency (F5D) is a rare hematological disorder with a variable spectrum of bleeding manifestations. Until now, no specific protocols for the management of these patients have been established. However, available literature suggests that perioperative infusion of fresh frozen plasma (FFP) may help maintain FV levels to prevent bleeding. We present the case of a 64-year-old man with previously undiagnosed severe FV deficiency and mild FV inhibitor, who underwent aortic valve replacement with no bleeding complications.

## Introduction

Factor V (FV) deficiency (F5D), also known as Owren’s disease or parahemophilia, is a rare autosomal recessive, hematological disorder, with an incidence of one case per one million people [1-4]. FV abnormalities can result in both hemorrhagic and thrombotic disorders, which makes FV unique among other coagulation factor deficiencies [5]. F5D patients may present with clinical manifestations ranging from mucosal bleeding to life-threatening hemorrhagic episodes [1]. Laboratory screening on these patients typically reveals prolonged prothrombin time (PT), activated partial thromboplastin time (aPTT), and a normal thrombin time (TT) [1]. To date, there are no well-established protocols for the management of these patients. No recombinant FV or plasma-derived FV concentrate is commercially available, and other factor concentrates, such as prothrombin complex concentrate, do not contain FV [6]. Pre-interventional infusion of fresh frozen plasma (FFP) has been shown to reduce bleeding in some of these patients [1-3,5-6]. We report a case of F5D, with superimposed mild FV inhibitor, who underwent aortic valve replacement (AVR) with minimal FFP infusions and no abnormal bleeding events.

## Case presentation

A 64-year-old South Asian gentleman, born out of a non-consanguineous marriage, with a history of aortic stenosis (AS) without coronary artery disease (CAD), presented with dyspnea on exertion to his primary care clinician. He was evaluated for potential AVR by minimally invasive cardiac surgery (MICS). Pre-operative laboratory assessment showed a prolonged PT 54.4 seconds (normal range: 12-15) and an elevated PTT of 197 seconds (normal range: 23-36 seconds). The complete metabolic panel was unremarkable, and a complete blood count was significant for hemoglobin level of 12.4 mg/dL. His fibrinogen level was 468 mg/dL (normal range: 150-400 mg/dL). These prolonged PT and aPTT prompted additional coagulation studies. Reptilase, TT, and dilute TT were all normal, excluding the presence of a direct thrombin inhibitor or significant dysfibrinogenemia. Testing for lupus anticoagulant was also negative. A mixing study was performed and showed near-complete correction of a PT, 16.3 seconds and complete correction of PTT, 33.6 seconds. No major prolongation of PT or PTT was noted upon incubation of the mixed sample for one hour at 37º C. Factor levels are shown in Table 1.

**Table 1:** Levels of coagulation factors

Coagulation factor	Value	Normal range (%)
Factor II	74%	75-135
Factor V	<1%	70-135
Factor VII	79%	75-140
Factor VIII	147%	60-150
Factor IX	104%	70-130
Factor X	77%	70-130
Factor XI	60%	75-145

No major inhibitor pattern was noted on dilutions performed for the factor levels. However, because FV was <1% and the PT mixing study showed less than expected correction for isolated F5D for the coagulation reagents used, a more sensitive FV inhibitor screen, performed with a 4:1 mixing study, and Bethesda titer were requested at a reference laboratory. FV inhibitor screen was positive; Bethesda titer was <1%, indicating the presence of low titer FV inhibitor activity. Review of previous medical records revealed a prolonged INR of 3.0 (normal range: <1.1) months prior to admission. While coagulation assessment was being performed, the patient received vitamin K, with no effect on coagulation studies. Despite the apparent longstanding abnormal coagulation status, the patient had previously undergone tooth and nail extractions, without unusual bleeding. There was no history of blood transfusions. He was cleared for AVR, and FFP was made available to maintain FV activity, in the event that excessive intra- or perioperative bleeding occurred. Four units of intraoperative FFP were given, and his estimated blood loss during the minimal invasive AVR was 500 mL. Additional two units of FFP were given two days post-operatively by the critical care team for a 1 g/dL drop in hemoglobin but with no clinical evidence of bleeding. The patient was discharged on day seven, and on six months follow-up, he had no evidence of bleeding.

## Discussion

FV plays an important role in coagulation cascade with both pro-coagulant and anticoagulant functions [3,5]. It is synthesized by the liver as a glycoprotein, with roughly 80% present in plasma and approximately 20% stored in the alpha granules of platelets [3,6]. The activation of FV is catalyzed by α-thrombin, produced in an independent activation pathway. Once FV is activated, factor Va (FVa) then activates factor Xa (FXa), which leads to the conversion of prothrombin into thrombin in the second phase of the coagulation cascade [6]. FV also modulates the anticoagulant pathway by downregulating the activity of Factor VIII [3,6-8]. F5D is a rare hematological disorder with an incidence of one case per 1,000,000 that can be caused by acquired inhibitors of FV, or by an autosomal recessive trait, most commonly in West Asian countries, where consanguineous marriages are more common [7]. To our knowledge, our patient was born out of a non-consanguineous marriage, without a significant family history for bleeding disorders. The symptoms of F5D vary widely. They range from no major bleeding diathesis to minor mucosal and soft tissue bleeding (i.e., epistaxis and hemarthroses) to life-threatening bleeding, such as gastrointestinal bleeding and intracranial bleeding [2-7,9]. Although our patient had a history of recent dental surgery and nail extractions, without abnormal bleeding, preoperative laboratory assessment revealed a prolonged PT and a PTT, and an FV level <1%.

Our patient was challenging from a diagnosis standpoint. It is well-established that a previous history of abnormal bleeds has discriminant power for the diagnosis of bleeding disorders [10]. Our patient had no significant bleeding history and had previously undergone tooth and nail extractions without unusual bleeding. FV was found to be essentially absent, which, in the context of no significant bleeding history, raised the possibility of an acquired FV inhibitor. Mixing studies did not support the presence of a strong FV inhibitor; however, PT corrected less than expected for F5D alone. Indeed, a more sensitive assay for the detection of FV inhibitors, involving a 4:1 mixing study was performed at a reference laboratory and suggested the presence of a weak FV inhibitor. The Bethesda titers were <1. Despite these abnormalities, surgical intervention was required, and clinical management decisions needed to be made. There is no literature to guide whether the strength of a Bethesda titer of an FV inhibitor predicts greater than expected bleeding in the context of AVR. Efforts to identify previous coagulation assessments for our patient revealed prior elevated INR months prior to presentation, supporting the diagnosis of a longstanding F5D with a likely superimposed weak FV inhibitor. It is possible that previous exposures to exogenous FV through previous transfusions led to the development of this weak inhibitor, a known phenomenon in FV deficiency patients [11]. In this F5D, bleeding does not correlate with FV inhibitor levels, nor the prolongation of PT, and PTT, factor activity, or the duration of the FV inhibitor [12]. F5D can be classified as mild when the FV activity is >5%, moderate 1% to 5%, and severe when it is <1% [2-3]. Our patient had severe F5D with no signs of bleeding. This could be explained as an existent disparity between the activity of plasma and platelet FV or due to coincidental thrombophilic mutations [9]. Some authors indicate that in patients with FV levels <1%, residual platelet FV may be crucial for maintaining adequate hemostasis, which also explains the discrepancy between the FV levels, and the variable bleeding manifestations [3,8]. Due to its very low incidence, no guidelines or protocols have been established to treat this unusual entity [5]. Our strategy was to improve FV levels prior to and after AVR, including the infusion of FFP at 15-20 mL/kg to control bleeding. FFP maintains FV activity at >25% to 30% in cases of invasive testing, bleeding or surgery [6]. Our patient received four units of FFP intraoperatively, and no excessive bleeding was reported. Some authors have suggested transfusion of FFP three to ten days postoperatively to maintain FV levels, and prevent bleeding events [5]. In our case, two days after the intervention his hemoglobin levels dropped to 9.8 mg/dL, despite the absence of any active bleeding two units of FFP were administered by the critical care team. Our patient was discharged home with no evidence of active bleeding or major complications.

## Conclusions

F5D remains a rare bleeding disorder that must be suspected in patients with prolonged PT, PTT, and normal TT. Major surgical procedures can be performed in those patients. The exact role and dosage of FFP remains to be determined for these challenging cases.
